# Ipsilateral femoral neck and shaft fractures fixation with proximal femoral nail antirotation II (PFNA II): technical note and cases series

**DOI:** 10.1186/s13018-019-1524-z

**Published:** 2020-01-20

**Authors:** Kuen-Ting Wu, Shih-Jie Lin, Ying-Chao Chou, Hsiang-Hen Cheng, Po-Chong Wen, Che-Han Lin, Wen-Ling Yeh

**Affiliations:** 1 Department of Orthopaedic Surgery, Chang Gung Memorial Hospital, Chiayi, Taiwan; 2 Department of Orthopaedic Surgery, Chang Gung Memorial Hospital, Taoyuan, Taiwan; 3 Department of Orthopaedic Surgery, Chang Gung Memorial Hospital, Linkou, Taiwan

**Keywords:** Ipsilateral femoral neck and shaft fractures, Proximal femoral nail, Reduction, Cephalomedullary nail

## Abstract

**Background:**

Combined ipsilateral femoral neck and shaft fractures are an uncommon type of fractures. A number of different implant options are available for the management of this injury. Two-device procedures were suggested because of the higher rate of malunion by single-device treatment. However, surgical treatment using a cephalomedullary nail is still an alternative option that provides better mechanical advantage and minimal invasion. This study details the technique of treating these pattern fractures with proximal femoral nail anti-rotation II (PFNA-II) to achieve an acceptable reduction in both fracture sites.

**Methods:**

Ten cases of ipsilateral femoral neck and shaft fractures under reduction by PFNA II were included and reviewed. A saw-bone model was also utilized to perform the detailed technique of reduction and fixation of PFNA II.

**Results:**

Under the special technique by using the PFNA II, all ten cases achieved optimal reduction and alignment of both fracture sites in intra-operative fluoroscopy. There was no intra-operative complication noted. After 6 months of follow-up, radiography revealed proper alignment and well union of the fractures.

**Conclusions:**

Fixation of ipsilateral femoral neck and shaft fractures with a single construct provides advantages of good biomechanical function, minimal invasion, reduced blood loss, and less operation time when comparing to two-device fixation. Thus, if acceptable reduction could be achieved, fixation by one PFNA II was a good alternative choice for this injury pattern.

## Background

Combined ipsilateral femoral neck and shaft fractures are an uncommon type of fractures. Associated ipsilateral femoral neck fractures have been reported to occur in as high as 10% of all femoral shaft fractures [1, 2]. In the past research, most surgeons have recommended fixation with two separate prostheses because the use of a single cephalomedullary device often results in higher rates of complication and malunion.

In spite of the lower rate of malreduction in two-device constructs, a non-traumatic fracture in the gapped area between two implant constructs in a patient with healed ipsilateral intertrochanteric and shaft fractures has been observed [3, 4]. Comparing to two-device techniques, fixation of both fractures with a single construct with either reconstructive or cephalomedullary nails provides advantages such as minimal invasion, reduced blood loss, and less operation time. In addition, there will be no gapped region and no concerned stress riser in a single nailing construct. Proximal femoral nail antirotation (PFNA) was designed initially for the fixation of peritrochanteric fractures while the second-generation nail was introduced specifically to accommodate the femoral size and geometry of Asians. Femoral neck fractures might be one of contraindication of this construct. However, among this specific injury cohorts, the basicervical type of femoral neck fracture is the most common with a range from 63% to 89% [5, 6], which is not the contraindication of PFNA II.

**Fig. 1:**
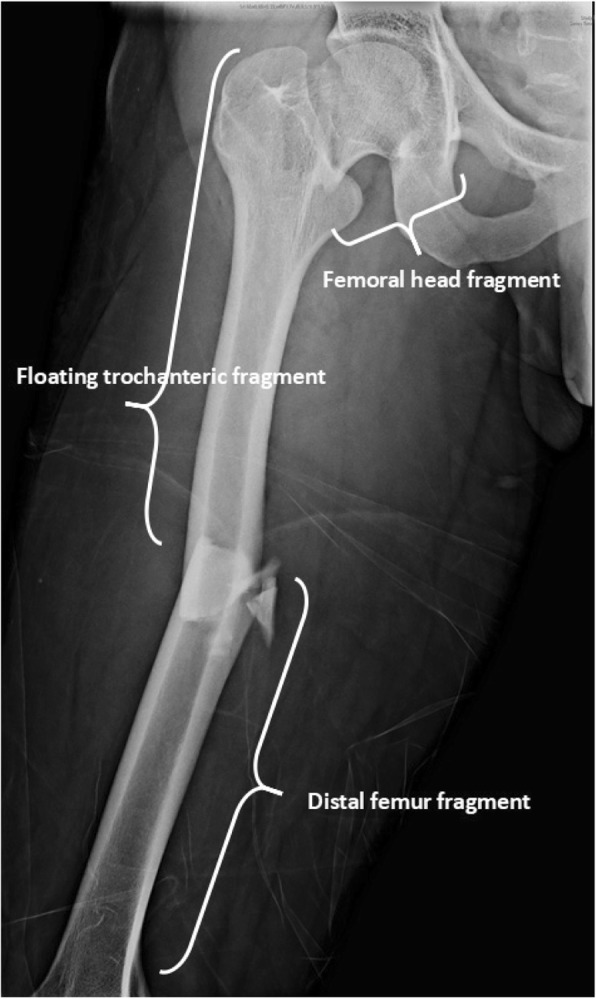
In ipsilateral femoral neck and shaft fractures, femur bone can be divided into three parts: femoral head fragment, floating trochanteric fragment, and distal femur fragment

However, difficulties in obtaining rotational alignment of nail and reduction of a neck fracture, especially, when the femoral neck injury is displaced were reported [7–9]. Using indirect reduction in closed antegrade/retrograde nailing may have mal-rotation in the shaft fracture. When using antegrade cephalomedullary nails to treat femoral neck and shaft fractures, the floating trochanteric fragment (Fig. 1) may easily cause malreduction in both fracture sites. Some reports have suggested substantial internal rotation of this floating fragment may be helpful during nailing and placement of cephalomedullary head screw [8].

To our knowledge, there were few reports discussing the use of PFNA II to treat the cohort with combined injuries. This study investigates the case series clinically and radiographically and presents the techniques treating ipsilateral femoral neck and shaft fractures with PFNA-II to improve optimal reduction in both fracture sites.

## Materials and methods

### Material

This is a retrospective case series in a single referral hospital. In this study, we reviewed the cases of the ipsilateral femoral neck and shaft fractures over a period of 2 years (August 2016 to August 2018) using a single implant of cephalomedullary nail fixation. The patients who underwent two-implant fixation or a single-implant fixation other than cephalomedullary nail fixation were excluded. We also excluded one of the patients in whom the proximal femoral fractures were initially missed and revised with a cephalomedullary fixation. After consideration of the inclusion and exclusion criteria, we present our surgical techniques in managing 10 subjects, 8 men and 2 women, aged between 18 and 67 years (median age, 29.5 years), who underwent internal fixation with single cephalomedullary nail for ipsilateral femoral neck and shaft fractures, met the inclusion criteria and were enrolled in this study. For each patient, the basic demographic characteristics were collected. Besides, radiographic findings for both fracture sites were also recorded. In our case series, we used the AO/OTA system and Winquist classification to describe the femoral shaft fractures. And for the femoral neck fractures, Garden classification and Pauwel’s classification were used to demonstrate the fracture pattern.

The device used is the proximal femoral nail anti-rotation II (PFNA-II; Synthes, Pennsylvania, USA; Synthes GmbH, Oberdorf, Switzerland). The PFNA-II is an intramedullary device designed for fixation of proximal femoral fractures involving trochanteric regions. Its anti-rotated helical blade can compact cancellous bone and thus increase stability against rotation and varus collapse [10]. This nail provides different nail lengths (standard, 240 mm; small, 200 mm; extra small, 170 mm; and long, 260–340 mm) with four different distal diameters (9, 10, 11, and 12 mm). With different nail lengths, it could be used to allow the stabilization of shaft fracture at any level.

## Methods

### Approach

The procedure is performed under a patient in supine on a fracture table as a regular closed nailing procedure. Traction force for the affected limb can be controlled by the distal support of boot on the foot. Abduct the unaffected leg as far as possible to allow free fluoroscopic examination.

Minimal invasion is one of the main aims in closed nailing. However, mini-open approach to the shaft fracture to achieve better anatomic reduction by facilitating guide wire passage or to rotate nail in an optimal position can be considered if necessary.

After a proper pre-operative position and reduction under fracture table, the entry point is determined on the tip of the greater trochanter and the guide wire is inserted through the shaft fracture site and deep into distal metaphyseal level. After inserting the guide wire in a suitable depth, medullary canal is prepared by reamed to a diameter 0.5 to 1.0 mm greater than the nail diameter.

### Pitfalls

While the PFNA-II nail is inserted into the medullary canal and adequate depth of the nail is reached, the optimal blade anteversion can be adjusted by the rotation of the targeting guide in the mediolateral view. In conventional techniques of treating a pertrochanteric fracture, the targeting guide was usually externally rotated to obtain adequate anteversion of blade position. However, in cases with an ipsilateral neck and shaft fractures of the femur, the inherent nature of floating trochanteric fragment may cause nail-shaft mal-position in axial dimension because of external rotation of floating fragment [8]. External rotation of targeting guide may lead to more externally rotated trochanteric fragment and loss reduction of the neck fracture site.

### Reduction of the neck fracture

Acceptable femoral neck reduction is an important issue to reduce the rate of malalignment, nonunion, and head necrosis. After PFNA nail is inserted to adequate depth of the medullary canal. There are two relationships of the nail and floating trochanteric fragment: nail-shaft engaged and non-engaged.

In the nail-shaft engaged group, a suitable diameter of the nail was chosen and the nail was advanced adequately; the nail and trochanteric part would be engaged as a fused component. Then, the rotation, abduction, or adduction of the targeting guide would reduce the neck fracture site under a joystick technique.

**Fig. 2:**
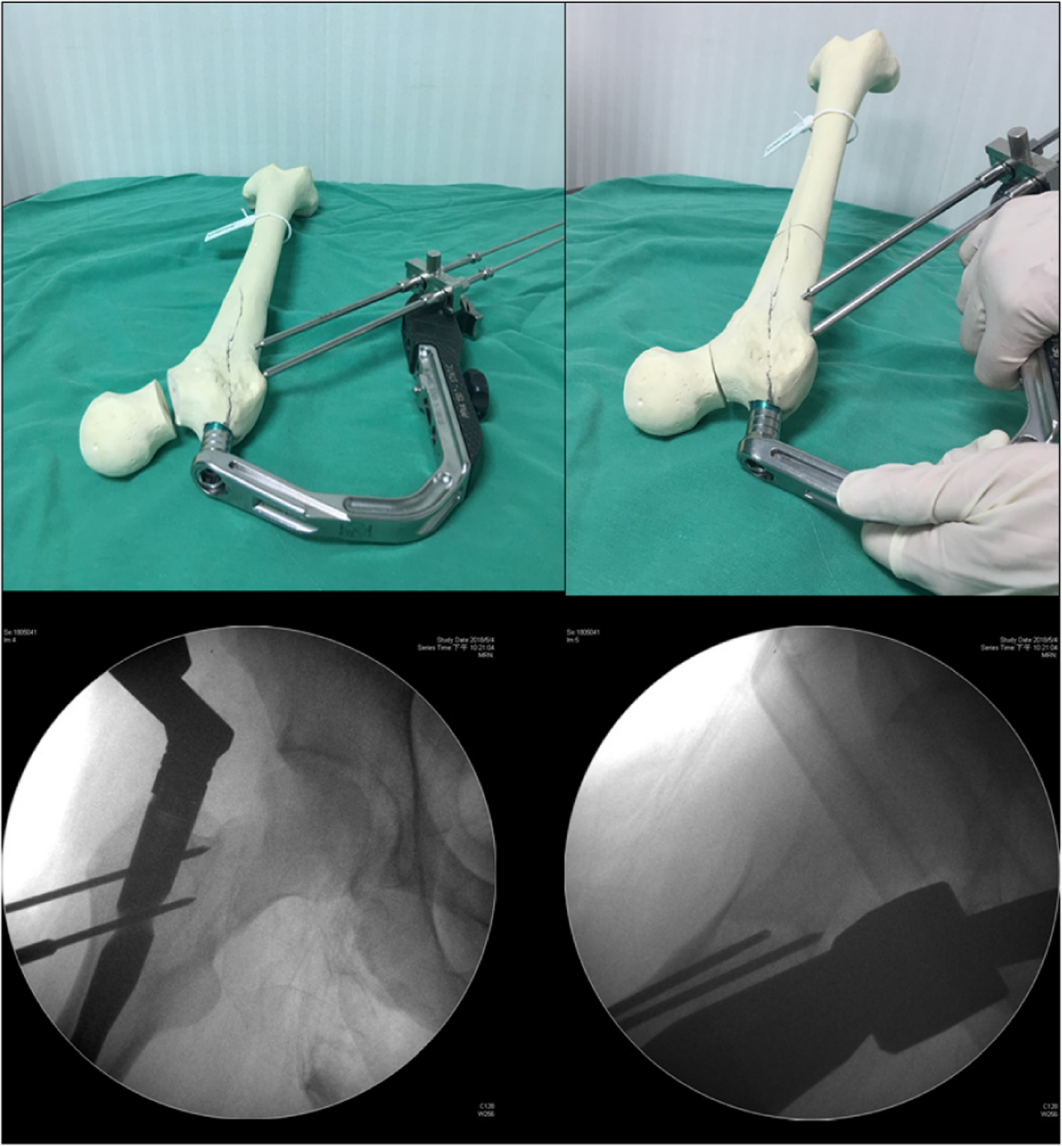
Aiming jig with two wires was inserted via drill sleeves to fix the floating trochanteric only and do not pass through the neck fracture line. And the target guide can be adjusted to achieve a good reduction of femoral neck fracture

However, in osteoporotic cases, no engagement of the nail-shaft construct will make the nail rotate easily in the floating trochanteric part while rotating the targeting guide. In this situation, following the joystick technique by the targeting guide, therefore, cannot be achieved. Under this condition, the aiming jig for anti-rotation wires should be used, and two wires were inserted via drill sleeves to fix the floating trochanteric fragment. The guide wire would be inserted to the proximal femur only and does not pass through the neck fracture line (Fig. 2). Then the PFNA nail and floating trochanteric part would be engaged.

After acceptable neck anatomic reduction was achieved by focal joysticks, two guide wires or K-pins can be placed to temporarily fix the neck at positions that do not hinder the nail. One anteriorly and another posteriorly or both posteriorly provides relative stability that can avoid subsequent dislodgement or rotation during further reposition of the nail to an optimal depth and anteversion.

### Restore nail-shaft alignment

In the nail-shaft engaged situation, repositioning of the nail is possible by partial retraction and readvancement of the nail. And in the nail-shaft non-engaged group, removing the previous guide wires and disconnecting the aiming jig makes the nail-shaft to non-engaged situation. Then the nail can be adjusted to an optimal anteversion. The anteversion can be determined by inserting a guide wire ventral to the femoral neck and head. After obtaining optimal guide wire position, drilling hole for blade and insertion of it by gentle blows with the hammer were performed.

### Reduction of shaft fracture

After proper reduction of femoral neck fracture, the distal femur part can be internally or externally rotated under dynamic view or mini-open reduction to achieve symmetric cortical thickening of the femur, which can reduce the rate of malalignment or impairment rotation function.

## Result

There were 10 cases of ipsilateral femoral neck and shaft fractures were involved from August 2016 to August 2018. There were 8 males and 2 females (4 right femur and 6 left femur) with age between 18 and 67 years (median age, 29.5 years). All patients were suffered from traffic accident events with 9 cases were soccer driver and 1 was trunk driver. All of the cases revealed good fracture reduction and after 12 months of follow-up, radiographs showed proper alignment and well union of both combined fractures.

In radiographic features, all of the femoral neck fractures were Pauwel’s type III, which the angle between fracture line and horizontal line on anteroposterior radiograph is greater than 50°. There were 7 displaced fractures (Garden types III and IV) and 3 non-displaced fractures (Garden type I, II). For the femur shaft fracture pattern, most (7 of 10) were simple fracture and three were wedge fractures; no comminuted fractures were noted. After closed reduction under fracture table or combined our technique for those retained displaced fractures noted, all the cases achieved satisfactory reduction in postoperative radiographs and recovered to a satisfactory union.

## Case presentation

### Case 1

**Fig. 3:**
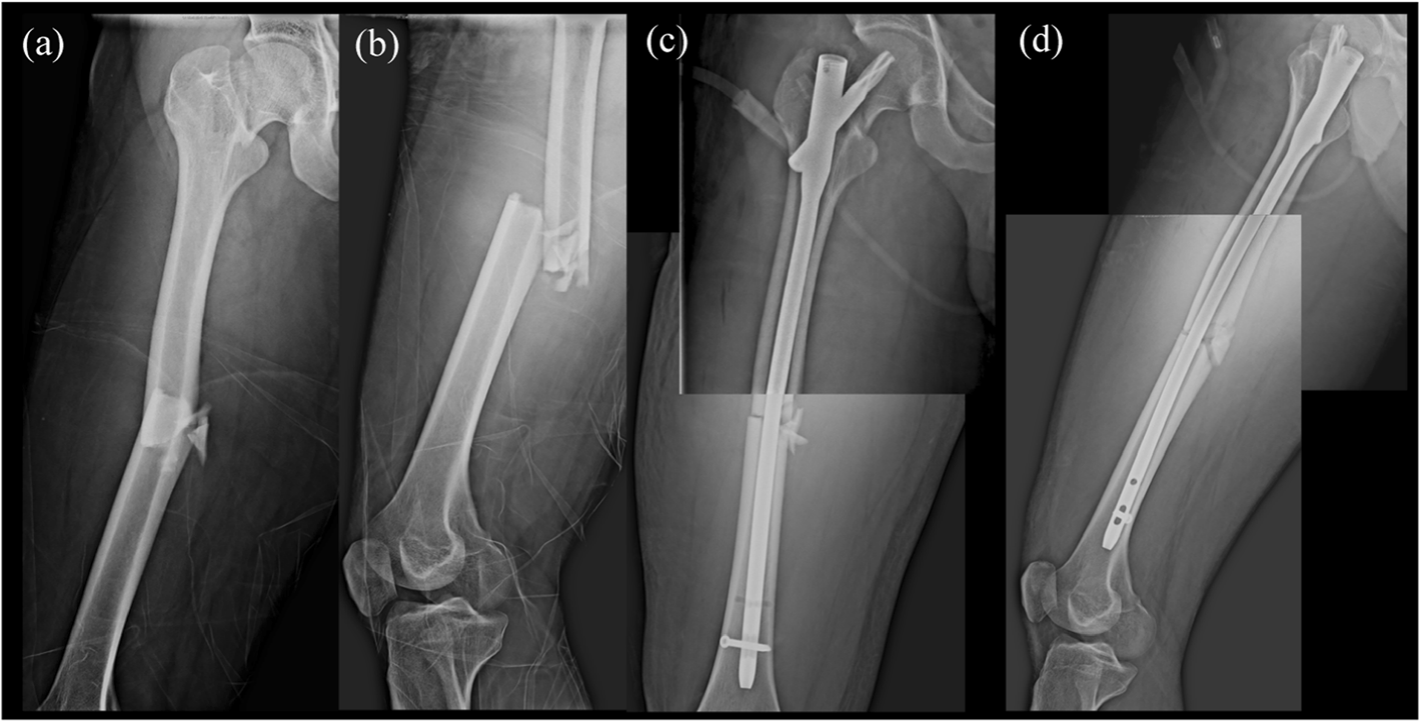
**a**,**b** Ipsilateral right femoral middle shaft fracture AO 32B3 and Garden type II, Pauwel’s classification type III (73′) femoral neck fracture. **c**, **d** ORIF with PFNA II (10 × 380 mm; blade, 95 mm)

The patient is a 27-year-old male who was suffered from a traffic accident that fell into the ditch from the motorcycle. He was brought to the emergency department with severe right thigh pain. Physical examination revealed right thigh tenderness and limited range of motion of the right hip. Radiography with a plain view of the right femur and CT of the abdomen showed ipsilateral femoral middle shaft fracture AO 32B3 and Garden type II, Pauwel’s classification type III (73°), AO31B2.3, femoral neck fracture (Fig. 3a, b).

Surgical intervention of open reduction and internal fixation (ORIF) with PFNA II (10 × 380 mm; blade, 95 mm) was performed. (Fig. 3c, d) Fluoroscopic view during operation revealed well alignment of both femoral neck and shaft fracture. After a few days of observation in ordinary ward, the patient was discharged successfully.

### Case 2

**Fig. 4:**
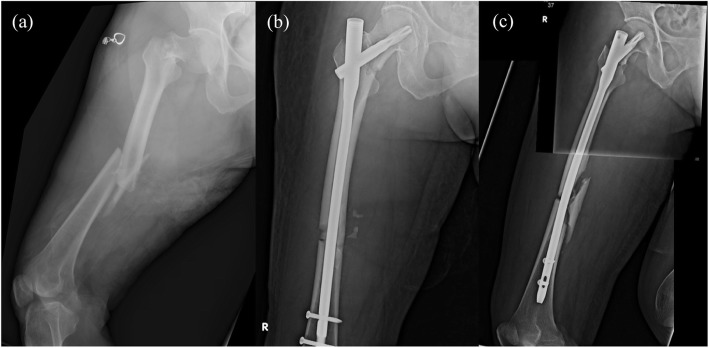
**a** Ipsilateral right femoral middle shaft fracture AO 32B2 and Garden type IV, Pauwel’s classification type III (65′) femoral neck fracture. **b**, **c** ORIF with PFNA II (10 × 340 mm)

The patient is a 67-year-old female who was a motorcyclist and crashed with a car. There was a loss of consciousness initially. She was brought to ER and an initial brain computed tomography scan revealed Subarachnoid hemorrhage at the right pre-pontine and Sylvian cistern. Besides, for right thigh deformity, radiography was performed and found the right femur middle shaft wedge fracture AO 32B2 with ipsilateral femoral neck fracture Garden type IV, AO31B2.3 and Pauwel’s classification type III (65°) (Fig. 4a).

**Fig. 5:**
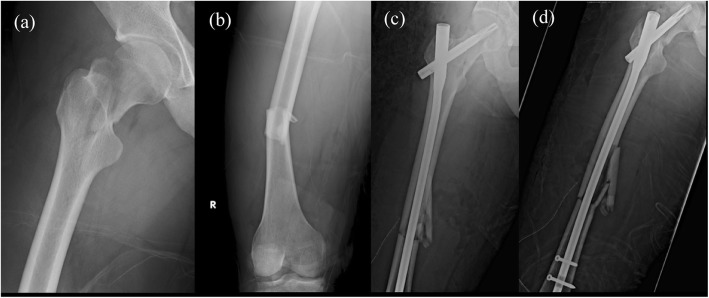
**a**, **b** Ipsilateral right femur middle shaft transverse fracture AO 32A3 with ipsilateral femoral neck fracture Garden type III and Pauwel’s classification type III (72′). **c**, **d** ORIF with PFNA II (10 × 380 mm; blade, 110 mm)

Due to the traumatic SAH, she was intubated and admitted to NSICU for further close observation and management. After her clinical condition was stable, ORIF with long PFNA II (10 × 340 mm) was performed on day 7 after the traffic accident. Fluoroscopic view during operation revealed well alignment of both femoral neck and shaft fracture (Fig. 4b, c).

### Case 3

The patient is a 30-year-old male who had traumatic event of traffic accident. Radiography and CT of the pelvis were arranged and showed the right femur middle shaft transverse fracture AO 32A3 with ipsilateral femoral neck fracture Garden type III and Pauwel’s classification type III (72°), AO31B2.3. Besides, the right patella subluxation was also found (Fig. 5a, b).

Surgical intervention of ORIF with PFNA II (10 × 380 mm; blade,110 mm) was performed for the ipsilateral femoral neck and shaft fracture on the day of the trauma. Fluoroscopic view during operation revealed well alignment of both femoral neck and shaft fracture (Fig. 5c,d).

## Discussion

Our present techniques put emphasis on achieving optimal reduction quality in the treatment of ipsilateral femoral neck and shaft fracture using PFNA-II. Some cases presented were associated with displaced neck fracture while being reduced under image intensifier. This can be extremely technically challenging [7, 9], but the present techniques would make the procedure smooth and the radiographic outcome satisfied.

When using two-device techniques, it was agreed that a prompt surgery with priority to stabilization of the femoral neck fracture in order to avoid the risk of further injury to femoral head blood supply [11]. However, some advocate for fixation of femoral shaft first to better control the leg during femoral neck reduction [12]. Whatever the reduction of neck fracture is still challenging due to the floating trochanteric fragment even when using a dynamic hip screw or cannulated screws. Malreduction would always be a critical issue in all implants fixing the neck fracture of this specific combined fracture pattern.

While timely surgical stabilization is now recommended in the treatment of ipsilateral femoral neck and shaft fractures, no consensus exists as to the most appropriate method of fixation for either fracture [12]. However, mal-reduction was reported in a 37-case series of Bedi et al., including one with neck malreduction and two with shaft malreduction using cephalomedullary device. The use of cephalomedullary device to fix both neck and shaft fractures resulted in a significantly higher mal-reduction rate comparing to the use of two devices [13]. Provisional fixation of the femoral neck with either a wire or a screw before nailing may prevent the displacement of the femoral neck fracture. However, the proximal part of PFNA-II is larger than that of conventional cephalomedullary device, and this technique maybe not feasible in using PFNA-II. We believed the present techniques could provide better neck reduction during nailing.

Watson and Moed [14] demonstrated using reconstruction-type intramedullary nail lead to an increased risk of nonunion. Biomechanically, the stability provided by reconstruction nails alone would be insufficient for intracapsular femoral neck fractures [15]. As the revolution of hardware designs, PFNA-II provides excellent rotation control and bone preservation based on its anti-rotated blade without reaming in mechanical tests [16, 17]. However, limited clinical data exist comparing helical the blade with lag screw [18].

The transcervical type of the femoral neck fracture is a contraindication for PFNA II due to the probability of rotation during fixation. However, from previous data review, most of the femoral neck fracture is basicervical type in ipsilateral femoral neck and shaft fractures group, which are not contraindicated of PFNA II. Bong-Ju et al. has retrospectively followed 19 patients with undisplaced femoral neck fracture treated by PFNA, and the bone union was achieved in 17 cases [19]. Moreover, Wang and his colleagues used the first-generation PFNA on patients with ipsilateral femoral neck and shaft fractures. This study reported that it was a good option and showed a fair outcome [20]. Besides, high grade of Pauwel’s classification femoral neck fractures is more common in this combined fracture pattern, which fixed-angle devices are more suitable for the neck fractures. Accordingly, as long as achieving good neck reduction, PFNA-II might be an implant choice to fix the femoral neck fracture. Besides, there were other advantages such as closed antegrade nailing with minimal exposure, reduced risk of injury to the femoral head blood supply, reduced perioperative blood loss, better biomechanical stability that allow early weight bearing, and excellent rotation control and bone preservation based on its anti-rotated blade design.

There is an emerging failed mode, medial cutout of PFNA-II in the treatment of hip fractures [21, 22]. The most important factor in reducing implant cutout of PFNA-II in fixation of pertrochanteric fracture was an adequate tip-apex distance of head screw [21]. Besides, neck-shaft malreduction was another modifiable significant risk factor for cephalomedullary nail cutout in the treatment of trochanteric fractures [23]. Especially, these increased failure rates in helical blade use have been shown in the basicervical femoral neck fractures [22, 24], although no single explanation for atypical cutout has been confirmed. The authors advocated the importance of fracture reduction in mitigating medial migration [21, 25].

No scientific study is available to definitively support any one method of stabilization over another for the treatment of ipsilateral femoral neck and shaft fracture [12]. Excellent reduction is the key to decrease the complication of femoral neck fracture in any surgical stabilizations. The current techniques may help surgeons obtain the optimal neck reduction of the challenging fracture pattern intra-operatively using PFNA-II or other cephalomedullary nail systems.

## Conclusion

Treatment options for ipsilateral femoral neck and shaft fractures vary among orthopedic surgeons. In current literature, low-level evidence suggests that separate femoral neck and shaft implants are more accepted and favored because of higher failure rates by using a single cephalomedullary nail. Optimal reduction in both fracture sites is a challenging reduction by one construct. In this series, special techniques by using PFNA II were performed in treating these cohorts with combined injuries. All cases showed a good reduction outcome. Further follow-up and comparative studies are needed to confirm the efficacy.

## Data Availability

The data of the present study is available from the corresponding author on request.

## Abbreviation

PFNA-II: Proximal femoral nail anti-rotation II
